# Appraisal of the costs, health effects, and cost-effectiveness of screening, prevention, treatment and policy-indicated evidence-based interventions for eating disorders: a systematic review protocol

**DOI:** 10.1186/s40337-023-00802-2

**Published:** 2023-05-24

**Authors:** Phillip Aouad, Moin Uddin Ahmed, Natasha Nassar, Jane Miskovic-Wheatley, Stephen Touyz, Sarah Maguire, Michelle Cunich

**Affiliations:** 1 MAINSTREAM Centre for Health System Research & Translation in Eating Disorders Collaboration, InsideOut Institute, University of Sydney, Sydney, NSW Australia; 2 InsideOut Institute, Faculty of Medicine and Health, University of Sydney and Sydney Local Health District, Sydney, NSW Australia; 3 Boden Initiative, Charles Perkins Centre, Faculty of Medicine and Health (Central Clinical School), University of Sydney, Sydney, NSW Australia; 4 Menzies Centre for Health Policy and Economics, Sydney School of Public Health, University of Sydney, Sydney, NSW Australia; 5 Sydney Local Health District, Sydney, NSW Australia; 6 Sydney Health Economics Collaborative, Sydney Local Health District, Camperdown, NSW Australia

**Keywords:** Eating disorder, Anorexia, Bulimia, Nervosa, Binge, Health economic, Evaluation, Cost, Burden

## Abstract

**Background:**

Having reliable information to make decisions about the allocation of healthcare resources is needed to improve well-being and quality-of-life of individuals with eating disorders (EDs). EDs are a main concern for healthcare administrators globally, particularly due to the severity of health effects, urgent and complex healthcare needs, and relatively high and long-term healthcare costs. A rigorous assessment of up-to-date health economic evidence on interventions for EDs is essential for informing decision-making in this area. To date, health economic reviews on this topic lack a comprehensive assessment of the underlying clinical utility, type and amount of resources used, and methodological quality of included economic evaluations. The current review aims to (1) detail the type of costs (direct and indirect), costing approaches, health effects, and cost-effectiveness of interventions for EDs; (2) assess the nature and quality of available evidence to provide meaningful insights into the health economics associated with EDs.

**Methods:**

All interventions for screening, prevention, treatment, and policy-based approaches for all Diagnostic and Statistics Manual (DSM-IV and DSM-5) listed EDs among children, adolescents, and adults will be included. A range of study designs will be considered, including randomised controlled trials, panel studies, cohort studies, and quasi-experimental trials. Economic evaluations will consider key outcomes, including type of resources used (time and valued in a currency), costs (direct and indirect), costing approach, health effects (clinical and quality-of-life), cost-effectiveness, economic summaries used, and reporting and quality assessments. Fifteen general academic and field-specific (psychology and economics) databases will be searched using subject headings and keywords that consolidate costs, health effects, cost-effectiveness and EDs. Quality of included clinical studies will be assessed using risk-of-bias tools. Reporting and quality of the economic studies will be assessed using the widely accepted Consolidated Health Economic Evaluation Reporting Standards and Quality of Health Economic Studies frameworks, with findings of the review presented in tables and narratively.

**Discussion:**

Results emanating from this systematic review are expected to highlight gaps in healthcare interventions/policy-focused approaches, under-estimates of the economic costs and disease-burden, potential under-utilisation of ED-related resources, and a pressing need for more complete health economic evaluations.

## Introduction

Health economics is a field of research that is focused on examining the associated value of behaviours and interactions with healthcare systems and healthcare consumption [1]. The “health economic impact” is an assessment of the efficacy, effectiveness and efficiency of interventions related to a wide range of health indices, most often disease-specific outcomes and quality-of-life [1, 2].

In Australia, it is conservatively estimated that over one million people are impacted by an eating disorder (ED) [3]; that is, 3 in 100 Australians (or 4% of the population) are currently dealing with the impact of a diagnosable ED [4]. Low prevalence rates may be attributable to the reluctance of individuals to present to healthcare facilities for EDs, particularly primary healthcare. While healthcare costs associated with EDs themselves are substantial, in the realm of billions for governments and thousands for individuals [5], engaging with ED care at a later stage-of-illness increases ED-related costs even further [6]. Specifically, data derived from the South Australian Health Omnibus Survey, 2017, estimated the overall cost of EDs is approximately $84 billion (due to years of life lost), and approximately $1.6 billion annually from lost earnings [7]. Globally in 2020, EDs had an estimated annual healthcare cost between approximately AUD 4900 to AUD 90,000 for Anorexia nervosa (AN); ~ AUD 1450 to ~ AUD 30,700 for Bulimia Nervosa (BN); and ~ AUD 2875 to ~ AUD 4700 for Binge Eating Disorder (BED) per patient [8], with a specific note made in Tannous and colleagues (2021) that these figures may be an over- or under- estimate given the self-reported utilisation of healthcare for ED treatment [7]. Moreover, these cost estimates are likely to be conservative at best, as population-level health economic analyses [5] [9] often overlook a range of costs related to the individuals with EDs, their family, wider community and health system, the community and economic differences that exist (e.g. Tannous et al., 2021)[7], or consider each of these factors in isolation—rather than the cost impact more broadly.

### Value-based personalised care

Conservative cost estimates for ED care may, in part, be attributable to the move in the health system to more personalised care and thus, the focus on measuring “value” based on what aspects of care are identified as important to individuals/patients and their families (i.e. individual values and preferences) [10]; leading to the measuring of personal, health system and societal costs/effects separately [11, 12]. For example, the resources used by individuals/patients to undertake a health intervention are recognised as meaningful costs such as costs to travel to a health facility, labour force/productivity losses and informal carer costs. Details on these exact items under the different categories, valuing them as opposed to reporting time losses only, and outlining the costing methods applied remain limited in published studies and reviews.

In a 2017 systematic review on the economic outcomes associated with the treatment of EDs [13], it was determined that ED interventions were largely cost-saving compared to other ED comparator groups (such as treatment as usual, placebo's, or waitlist) [13]. Some interventions such as cognitive dissonance therapy required a 90% participation rate for the course of treatment to be considered cost-saving [13] which, over the long-term, may not be entirely feasible. As such, long-term cost-effectiveness was unable to be determined [13].

While more specific research on the economic impact of ED intervention strategies is needed, the field lacks comprehensive economic assessments that take into consideration the increasing rate of ED research output, particularly in the last 5-years [13]; which can provide insights into new cost-effective interventions to prevent and treat EDs. The increase in ED research in recent years can be evidenced by the number of records returned during a preliminary literature search. Search results yielded a similar number of records between 2000–2017 and 2017–2022, despite a five-year vs two-decade difference in timeframe. This highlights that research into the health economic evaluation of ED interventions needs to take into consideration that recent research has increased almost exponentially. In short, what we do not know is how recent research and findings in the ED intervention space influence the economic effectiveness, outcomes, and direction used to inform decision-making by policy-makers and healthcare administrators.

Whilst reviews, such as the one conducted by Le and colleagues [13], have provided some assessment of the quality of the health economic evidence, they are limited in unpacking the resource use (cost) items in more detail; or undertaking a rigorous appraisal of the methodological quality of economic evaluations on health interventions for EDs. Therefore, a comprehensive examination of the broader ED health economic landscape in Australia and other “westernised, educated, industrialised, rich, and democratic” (WEIRD) [17] countries is critically needed to bring attention to the areas most impacted by the health economic cost of EDs and to inform policy, practice and service development.

### Research question, aims, and objectives

Specifically, the review will answer the following research question: What are the healthcare and other resources used, their economic costs, main health effects and cost-effectiveness, in the screening, prevention and treatment of, and policy-based approaches to, EDs in any patient age group?

Therefore, the proposed review aims to:

1. Detail the variety of costs broken down into direct [medical] and indirect costs, costing approaches, health effects and cost-effectiveness of interventions for EDs compared to standard care or a do-nothing scenario.
2. Evaluate the nature and quality of the evidence and sub-group analyses (data permitting) to develop a comprehensive picture of the health economic evidence associated with EDs, including health economic evidence on interventions for the screening, prevention, treatment, and policy-based approaches of EDs.

## Methods

This protocol is developed based on “The Preferred Reporting Items for Systematic Reviews and Meta-Analyses Protocols” (PRISMA-P) [14], with the protocol registered in PROSPERO (ID: CRD42022339694). The review itself will be conducted systematically, adhering to the current protocol and widely accepted PRISMA guidelines [15]. Given the need to generate a comprehensive picture of the health economic impacts of EDs worldwide, the review will be kept broad examining literature that takes into consideration both DSM-IV and DSM-5 listed EDs; there will be no restrictions set on the age or gender of study participants, or on the mode of intervention/service delivery (online and other forms of virtual healthcare, face-to-face).

### Study eligibility

To be considered eligible for inclusion in this systematic review, studies will need to incorporate the criteria outlined below:

### Inclusion criteria

The inclusion criteria are summarised in Table 1. Economic evaluation studies published in any language will be included and translation services used where applicable. Full economic evaluations, including cost-effectiveness analysis (CEA), cost–benefit analysis (CBA), and cost-utility analysis (CUA), will be considered in the review. Any intervention will be eligible for inclusion irrespective of the mode of intervention/service delivery, study setting, design of the clinical study, and country. This review will consider studies that compare the ED intervention or policy to an alternative standard of care or a do-nothing scenario.

**Table 1:** Study inclusion criteria

Sample population	Individuals with a DSM-IV or DSM-5 diagnoses specified eating disorder; carers or consumers of ED services
Types of intervention	All types of policy-indicated evidence-based interventions including but not limited to screening, prevention, treatment and policy-indicated evidence-based interventions for EDs– including psychotherapies, pharmacological and dietetic interventions
Participant age	Unrestricted
Condition	Must have a current or lifetime history of a diagnosed DSM-IV or DSM-5 eating disorder
Study type & design	All types—including but not limited to studies which undertake either trial-based comparative economic analyses or modelled economic analyses
Outcome measure	*Full economic evaluations* (see *Outcomes—Cost Measures for Health Economic Evaluations*, below)
Setting	Any. Such as clinical settings, inpatient, community, online and other types of virtual care samples
Country of study	Any. Including standard grouping such as World Bank high-income country groupings [16] or WEIRD (westernised, educated, industrialised, rich, democratic) countries [17]
Date of study	Only restricting the start date to 1994 to cover both DSM-IV and DSM-5 circulation periods
Publication type & availability	Peer-Reviewed, Full-Text Only
Language	Any

### Types of Studies

Study designs for trial-based economic evaluations will include randomised control trials (RCTs), non-randomised studies (NRS; non-randomised controlled trials), quasi-experimental, panel and repeated measures studies, pre- and post-intervention studies, prospective and retrospective cohort studies, and analytical cross-sectional studies.

### Phenomena of Interest

The current review will consider all EDs as listed in the DSM-IV and DSM-5, which consists of Anorexia nervosa, Bulimia Nervosa, Binge Eating Disorder, Eating Disorder Not Otherwise Specified (EDNOS), Other Specified Feeding and Eating Disorder (OSFED) (including listed phenotypes), Unspecified Feeding and Eating Disorder (UFED), Avoidant/restrictive food intake disorder (ARFID), Purging disorder, Rumination, Pica, Night Eating Syndrome. All full health economic evaluations will be considered in the review including cost-minimisation, cost-effectiveness, cost–benefit, and cost-utility analyses.

### Outcomes—Cost Measures for Health Economic Evaluations

This review will examine a wide range of resources used to develop, implement or take up from the patient’s perspective, and maintain interventions for EDs [18]. This will include reporting on the different perspectives used; costing approaches; and cost differentials between the various alternatives considered in the studies.

### Exclusion criteria

Descriptive economic studies will be excluded, studies not related to the health economic outcomes of EDs, as well as imperfect, or partial health economic evaluations. Specifically, studies that only report on the cost of interventions, burden of disease, cost-of-illness or do not conduct comparative economic evaluation (i.e., do not examine these costs in relation to study outcomes) will be excluded. Similarly, studies that do not specifically aim to examine the complete health economic impact of interventions for EDs will be excluded. Thus, studies that report only on the health effects or quality-of-life without the associated resource use (costs) will be excluded. Further, commentaries, editorials, study protocols, restricted works such as abstracts for conferences, or methodological articles will not be included. However, reference lists of such works, if relevant, will be examined to find further suitable studies for inclusion.

### Search strategy

The proposed search strategy will primarily involve intensive searching for peer-reviewed work/full reports on health economic evaluations of EDs. The search strategy will commence with a preliminary search using PubMed and EconLit to establish appropriate keywords (from the title, abstract, and indexed keywords) and ensure search terms will be accurate in capturing the required literature. This will be followed by searching identified keywords and indexed terms across all listed databases; and search strings across different databases reviewed by an experienced librarian. Once included studies have been established (screening title, abstract, and full-text as required), reference lists of studies will be searched by hand, first by title, then abstract, then full-text if required—which may help locate extra peer-reviewed, but not indexed, studies.

### Databases and reference management

An electronic search will be conducted on the following databases from inception to the present date (expected: August 2022): MEDLINE; Embase; Cochrane; PsycInfo; Global Health; NHS Economic Evaluation Database; EBM Reviews—Health Technology Assessment; ERIC; CINAHL; Academic Search Complete; Health Business Elite; EconLit; Scopus; The Cost Effectiveness Analysis (CEA) Registry; and Paediatric Economic Database Evaluation (PEDE). Search results will be stored in Endnote version 20 (current version: 20; Clarivate Plc). After removing the duplicate records from the database, citations will be imported into Rayyan [19], where the studies will be screened.

### Selection process

Studies will be screened by two independent researchers (MC and MUA) to determine their eligibility for inclusion in the review. Full text studies will be retrieved in the absence of sufficient information in the titles and abstracts. After this, full-text articles will be screened by two independent researchers (MC and MUA) for inclusion in the review. Disagreements will be discussed and if required mediated or determined by a third independent researcher. The reasons for exclusion will also be recorded. The result of the study selection process will be presented in the PRISMA flow diagram. 

### Data collection and extraction

From included studies, data extraction will be performed using the Microsoft Excel program. Data to be extracted will include bibliographic and study details including main aims; economic evaluation type (RCT-based or a modelled study); population; setting of the study; disease-specific health effects; more general health effects; interventions; resources used and associated costs; health economic analysis conducted (outcomes of the economic studies); and findings and conclusions [20].Data will be extracted based in part on the standardised data extraction protocol outlined in the Joanna Brigs Institute Reviewers Manual 2014 for the Systematic Review of Economic Evaluation Evidence (JBI ACTUARI) [21].

### Selection of studies, data analysis and synthesis

Where possible, data will be pooled using the JBI ACTUARI guidelines and summaries provided in a table format. If economic and other data are unable to be pooled or presented in a table, it will be presented in narrative form. A narrative-type summary will be used to report findings of the review.

### Quality and risk of bias assessment

The reporting quality of the economic evaluation studies will be assessed using the Consolidated Health Economic Evaluation Reporting Standards (CHEERS) statement [22], whereas the methodological quality will be assessed using the Quality of Health Economic Studies (QHES) list [23]. Risk of bias will be assessed using the ROBINS-I tool (Risk of bias in non-randomized studies-of interventions) [24] and RoB 2 tool (revised tool for Cochrane’s Risk of Bias in randomized trials) [25]. Reviewers will take particular note of reporting biases including publication bias, time lag bias, outcome reporting bias, and duplicate publication bias.

## Discussion

The proposed systematic review will assess the health economic impacts of interventions for EDs worldwide. Classification of interventions; differences in costs, health effects and health economic indices as well as their implications for the management of EDs within reasonable country groupings will be examined. The findings of the proposed systematic review will provide the foundation for recommendations to improve both the EDs treatment landscape and aid in understanding more widely the benefits of early interventions.

**Fig. 1:**
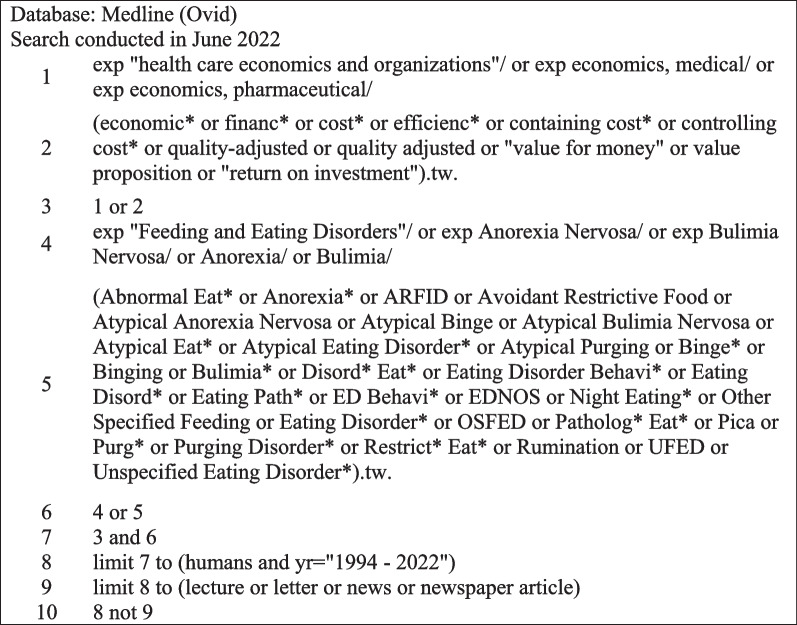
MEDLINE Search Method

Preliminary searching of four main databases using the search string outlined resulted in ~ 16,000 initial records (prior to any form of screening and remove of duplicates). For example, the search method utilised for MEDLINE (Ovid platform), see Fig. 1, yielded 3600 records; Web of Science, ~ 4500; Embase, ~ 4800; and Academic Search Complete, ~ 3300.

### Expected limitations and strengths

Generally, it is expected that there will be limitations due to the types of studies that meet eligibility criteria, thus, restricting the conclusions able to be drawn. Given the differences between health systems in the group of countries considered in this proposed review, it is likely that findings/databases may, at times, be difficult to interpret. However, due to the collaborative nature of the proposed review, specialist health economic knowledge will help determine if studies have conducted partial or full economic analysis. Additionally, studies that are not written in English can be included by utilising Google Translate or interpretation services if required. Overall, the expected challenges may, in part, limit generalisability of results as conclusions may not be applicable to all situations or health systems and therefore, outcomes will need to be interpreted with care.

A primary strength of the proposed systematic review is the utilisation of a comprehensive search strategy that spans intervention more widely than ever before. Additionally, the inclusion of a broad range of major databases in medicine, public health/policy, and psychology as well as subject-specific databases (e.g., Health Technology Assessment and EconLit) add to the likelihood of capturing studies that may otherwise be missed. The review will also perform a more thorough analysis of the resource used and associated costs including detailing the categories of costs measured and break these down into direct or indirect for the range of interventions considered (i.e. screening, prevention, treatment and policy); perspective taken (individuals/patients, health system, society as a whole); whether the resource used were measured in natural units (e.g., time) or valued in a currency; and the valuation method used. It will be informative to identify how the costs compare for the different types of interventions for EDs; what cost categories have attributed to most of the total costs for screening, prevention, treatment, and policy interventions separately; and the areas where there may be cost efficiencies to be had.

The proposed review will be the first comprehensive examination of the health economic impact associated with interventions for EDs. It is expected that the findings will help to inform guidelines, policy and develop a more harmonised approach to undertaking health economic evaluations in this clinical area.

## Data Availability

Not applicable.

## Abbreviations

AN: Anorexia nervosa
ARFID: Avoidant/restrictive food intake disorder
BED: Binge eating disorder
BN: Bulimia nervosa
CBA: Cost–benefit analysis
CEA: Cost-effectiveness analysis
CHEERS: Consolidated Health Economic Evaluation Reporting Standards
CUA: Cost-utility analysis
DSM: Diagnostic and statistics manual (American Psychiatric Association)
ED: Eating disorder
EDNOS: Eating disorder not otherwise specified
MRFF: Medical Research Future Fund
NRS: Non-randomised studies (non-randomised controlled trials)
OSFED: Other specified feeding and eating disorder
PRISMA: Preferred reporting items for systematic reviews and meta-analyses
QHES: Quality of Health Economic Studies
RCT: Randomised control trials
ROB2: Risk of bias in randomized trials
ROBINS-I: Risk of bias in non-randomized studies-of interventions
UFED: Unspecified feeding and eating disorder
WEIRD: Westernised, educated, industrialised, rich, democratic

## References

[1] Meltzer MI (2001). Introduction to health economics for physicians. Lancet.

[2] Drummond MF, Sculpher MJ, Claxton K, Stoddart GL, Torrance GW (2015). Methods for the economic evaluation of health care programmes.

[3] Wade TD, Bergin JL, Tiggemann M, Bulik CM, Fairburn CG (2006). Prevalence and long-term course of lifetime eating disorders in an adult Australian twin cohort. Aust N Z J Psychiatry.

[4] Hay P, Mitchison D, Collado AEL, González-Chica DA, Stocks N, Touyz S (2017). Burden and health-related quality of life of eating disorders, including Avoidant/Restrictive Food Intake Disorder (ARFID), in the Australian population. J Eat Disord.

[5] Paxton SJ, Hay P, Touyz SW, Forbes D, Madden S, Girosi F, et al. Paying the price: the economic and social impact of eating disorders in Australia. 2012.

[6] Gatt L, Jan S, Mondraty N, Horsfield S, Hart S, Russell J (2014). The household economic burden of eating disorders and adherence to treatment in Australia. BMC Psychiatry.

[7] Tannous WK, Hay P, Girosi F, Heriseanu AI, Ahmed MU, Touyz S (2021). The economic cost of bulimia nervosa and binge eating disorder: a population-based study. Psychol Med.

[8] Ágh T, Kovács G, Supina D, Pawaskar M, Herman BK, Vokó Z (2016). A systematic review of the health-related quality of life and economic burdens of anorexia nervosa, bulimia nervosa, and binge eating disorder. Eat Weight Disord Stud Anorex Bulim Obes.

[9] Deloitte Access Economics. The Social and economic cost of eating disorders in the United States of America: a report for the strategic training initiative for the prevention of eating disorders and the academy for eating disorders. 2020.

[10] Rother J (2017). Top of the administration's agenda: stem the rising cost of healthcare. Generations.

[11] Dowie J, Kjer Kaltoft M, Salkeld G, Cunich M (2015). Towards generic online multicriteria decision support in patient-centred health care. Health Expect.

[12] Salkeld G, Cunich M, Dowie J, Howard K, Patel MI, Mann G (2016). The role of personalised choice in decision support: a randomized controlled trial of an online decision aid for prostate cancer screening. PLoS ONE.

[13] Le LK-D, Hay P, Mihalopoulos C (2018). A systematic review of cost-effectiveness studies of prevention and treatment for eating disorders. Aust N Z J Psychiatry.

[14] Shamseer L, Moher D, Clarke M, Ghersi D, Liberati A, Petticrew M (2015). Preferred reporting items for systematic review and meta-analysis protocols (PRISMA-P) 2015: elaboration and explanation. BMJ Br Med J.

[15] Moher D, Liberati A, Tetzlaff J, Altman DG (2009). Preferred reporting items for systematic reviews and meta-analyses: the PRISMA statement. Ann Intern Med.

[16] The World Bank. World Bank Country and Lending Groups 2022 [14 June 2022]. Available from: https://datahelpdesk.worldbank.org/knowledgebase/articles/906519-world-bank-country-and-lending-groups.

[17] Henrich J, Heine SJ, Norenzayan A (2010). The weirdest people in the world?. Behav Brain Sci.

[18] Coast J, Bailey C, Kinghorn P (2018). Patient centered outcome measurement in health economics: Beyond EQ-5D and the quality-adjusted life-year-where are we now?. Ann Palliat Med.

[19] Ouzzani M, Hammady H, Fedorowicz Z, Elmagarmid A (2016). Rayyan—a web and mobile app for systematic reviews. Syst Rev.

[20] Luhnen M, Prediger B, Neugebauer EA, Mathes T (2017). Systematic reviews of health economic evaluations: a protocol for a systematic review of characteristics and methods applied. Syst Rev.

[21] Joanna Briggs Institute. Joanna Briggs institute reviewers manual 2014 for the systematic review of economic evaluation evidence (JBI ACTUARI). Adelaide: The Joanna Briggs Institute; 2014.

[22] Husereau D, Drummond M, Petrou S, Carswell C, Moher D, Greenberg D (2013). Consolidated health economic evaluation reporting standards (CHEERS)—explanation and elaboration: a report of the ISPOR health economic evaluation publication guidelines good reporting practices task force. Value Health.

[23] Chiou C-F, Hay JW, Wallace JF, Bloom BS, Neumann PJ, Sullivan SD (2003). Development and validation of a grading system for the quality of cost-effectiveness studies. Med Care.

[24] Sterne JAC, Hernán MA, Reeves BC, Savović J, Berkman ND, Viswanathan M (2016). ROBINS-I: a tool for assessing risk of bias in non-randomised studies of interventions. BMJ.

[25] Sterne JAC, Savović J, Page MJ, Elbers RG, Blencowe NS, Boutron I (2019). RoB 2: a revised tool for assessing risk of bias in randomised trials. BMJ.

